# CD36 is indispensable for nutrient homeostasis and endurance exercise capacity during prolonged fasting

**DOI:** 10.14814/phy2.13884

**Published:** 2018-10-07

**Authors:** Tatsuya Iso, Hikari Haruyama, Hiroaki Sunaga, Hiroki Matsui, Miki Matsui, Rina Tanaka, Yogi Umbarawan, Mas Rizky A. A. Syamsunarno, Mirasari Putri, Aiko Yamaguchi, Hirofumi Hanaoka, Kazuaki Negishi, Tomoyuki Yokoyama, Masahiko Kurabayashi

**Affiliations:** ^1^ Department of Cardiovascular Medicine Gunma University Graduate School of Medicine Maebashi Gunma Japan; ^2^ Department of Laboratory Sciences Gunma University Graduate School of Health Sciences Maebashi Gunma Japan; ^3^ Department of Internal Medicine Faculty of Medicine Universitas Indonesia Jakarta Indonesia; ^4^ Department of Biochemistry and Molecular Biology Universitas Padjadjaran Jatinangor West Java Indonesia; ^5^ Department of Public Health Gunma University Graduate School of Medicine Maebashi Gunma Japan; ^6^ Department of Biochemistry Universitas Islam Bandung Bandung Indonesia; ^7^ Department of Bioimaging Information Analysis Gunma University Graduate School of Medicine Maebashi Gunma Japan; ^8^ Department of Cardiovascular Imaging Menzies Institute for Medical Research University of Tasmania Hobart Australia; ^9^ Nepean Clinical School University of Sydney Kingswood NSW Australia

**Keywords:** Exercise physiology, fasting, fatty acid, glucose, liver, metabolism, skeletal muscle

## Abstract

During fasting, most tissues including skeletal muscle heavily rely on utilization of fatty acids (FA) and minimize glucose use. In contrast, skeletal muscle prefers carbohydrate use as exercise intensity increases. In mice deficient for CD36 (CD36^−/−^ mice), FA uptake is markedly reduced with a compensatory increase in glucose uptake in skeletal muscle even during fasting. In this study, we questioned how exercise endurance is affected during prolonged fasting in CD36^−/−^ mice where glucose utilization is constantly increased. With or without a 24‐h fast, a single bout of treadmill exercise was started at the speed of 10 m/min, and the speed was progressively increased up to 30 m/min until mice were exhausted. Running distance of wild type (WT) and CD36^−/−^ mice was comparable in the fed state whereas that of CD36^−/−^ mice was significantly reduced after a 24‐h fast. Glycogen levels in liver and skeletal muscle were depleted both in WT and CD36^−/−^ mice after a 24‐h fast. In CD36^−/−^ mice, FA uptake by skeletal muscle continued to be reduced during fasting. Glucose utilization also continued to be enhanced in the heart and oxidative skeletal muscle and glucose supply relative to its demand was diminished, resulting in accelerated hypoglycemia. Consequently, available energy substrates from serum and in muscle for exercise performance were very limited in CD36^−/−^ mice during prolonged fasting, which could cause a remarkable reduction in exercise endurance. In conclusion, our study underscores the importance of CD36 for nutrient homeostasis to maintain exercise performance of skeletal muscle when nutrient supply is limited.

## Introduction

Energetically, exercise and fasting are both in catabolic states, but show distinct fuel selection. Exercise prefers carbohydrate use as exercise intensity increases, whereas fasting promotes consumption of more lipids.

In skeletal muscle, carbohydrates and lipids are oxidized to provide high‐energy phosphate (adenosine triphosphate, ATP) at rest and during exercise. During exercise, the absolute power output determines the rate of ATP demand and energy expenditure, whereas exercise intensity influences the relative contributions of carbohydrate and lipid sources and circulating (extramuscular) and intramuscular fuel stores, to energy provision (Egan and Zierath 2013; Yoshida et al. 2013). At low‐to‐moderate intensities of exercise, the primary fuel sources supplying skeletal muscle are glucose, derived from liver (glycogenolysis or gluconeogenesis) or oral ingestion, and non‐esterified fatty acids (NEFA) liberated by adipose tissue lipolysis. As exercise intensity increases, muscle utilization of circulating NEFA declines modestly, whereas utilization of circulating glucose increases progressively up to near‐maximal intensities. This coincides with increasing contribution of muscle glycogen to energy provision and absolute rates of carbohydrate oxidation with a majority of energy although reliance on muscle glycogen for sustained exercise is different between humans and mice (Pederson et al. 2005a,b). Conversely, intramuscular triglycerides (TG) constitute only a fraction (1–2%) of whole‐body lipid stores. When exercise at a fixed moderate intensity is prolonged (>60 min), energy contribution of lipid oxidation of intramuscular TG is increased. Thus, high‐intensity exercise increases the relative contributions of carbohydrate to energy provision while low‐to‐moderate exercise facilitates consumption of both carbohydrate and lipid as energy substrates.

During prolonged fasting, most tissues heavily rely on utilization of fatty acids (FA) while glucose use is minimized except brain and red blood cells (Salway 2004; Marray 2009). Prolonged fasting promotes the hydrolysis of TG in adipose tissue, thereby increasing the plasma concentration of NEFA, which is crucial energy substrate for peripheral tissues including skeletal muscle. As fasting is prolonged, glycogen storage in liver and skeletal muscle becomes empty while liver gluconeogenesis is accelerated to maintain blood glucose levels (Salway 2004; Marray 2009). Because liver and skeletal muscle glycogen is closely associated with exercise performance, especially for high‐intensity exercise, prolonged fasting causes early exhaustion for strenuous exercise (Pederson et al. 2005a,b; Egan and Zierath 2013).

Fatty acid translocase (FAT/CD36) is a single‐chain 88‐kDa glycoprotein that has wide biological functions in various kinds of cells, such as white and brown adipocytes, monocyte/macrophage and cardiac and skeletal muscle (Glatz et al. 2010). It has the capacity to be involved in facilitating long‐chain FA transport into adipocytes and cardiac and skeletal muscle. Under resting conditions, CD36^−/−^ mice display reduced rates of skeletal muscle FA transport and oxidation (Coburn et al. 2000; Hajri et al. 2002). To compensate for reduced FA utilization, CD36^−/−^ animals exhibit marked enhancement of glucose use with greater muscle insulin sensitivity (Coburn et al. 2000; Hajri et al. 2002). CD36^−/−^ mice also exhibit reduced exercise performance in the case of acute bout exercising at 78% *V*O2max (submaximal intensity), which was accompanied by considerably greater carbohydrate utilization and early depletion of hepatic glycogen despite of no prior fasting (McFarlan et al. 2012). Since the depletion of hepatic glycogen has long been linked with an impaired exercise performance, it is thought that the accelerated and almost complete hepatic glycogen depletion in CD36^−/−^ mice accounted for early exhaustion.

In this study, we further questioned whether exercise endurance is more severely affected in CD36^−/−^ mice during prolonged fasting, when glycogen storage in liver and skeletal muscle are already depleted prior to exercise. In other words, we tested whether uptake of energy substrates from circulation influences exercise performance irrespective of liver and muscle glycogen storage. We found that FA uptake by red/oxidative skeletal muscle and blood glucose levels were severely decreased during fasting, which could cause reduced endurance capacity in CD36^−/−^ mice. Our study underscores the importance of CD36 for nutrient homeostasis to maintain exercise performance of skeletal muscle when nutrient supply is limited.

## Materials and Methods

### Mice and sample collection

Mice with a homozygous null mutation in CD36 were generated as previously described (Febbraio et al. 1999). Control male wild‐type (WT) C57BL6j mice were purchased from Japan SLC, Inc. The ages (10–12 weeks) and body weights (22–27 g) of WT and CD36^−/−^ mice were comparable for all experiments. All study protocols were approved by The Institutional Animal Care and Use Committee (Gunma University Graduate School of Medicine). The mice were housed in a temperature‐controlled room in a 12‐h light/12‐h dark cycle and had unrestricted access to water and standard chow (CE‐2, Clea Japan, Inc.). For the fasting experiments, the mice were individually housed and food was withdrawn for 24 h; water was provided ad libitum. Blood was collected from retro‐orbital plexus to measure biochemical parameters and centrifuged at 1500*g* for 15 min at 4°C to separate the serum. Samples of liver, skeletal muscle, and heart were snapped frozen in liquid nitrogen and conserved at −80°C until further use.

### Biodistribution of ^125^I‐BMIPP (15‐(p‐iodophenyl)‐3‐(R,S)‐methyl pentadecanoic acid) and ^18^F‐FDG (2‐fluorodeoxyglucose)

Biodistribution of ^125^I‐BMIPP and ^18^F‐FDG was determined as described previously (Iso et al. 2013; Syamsunarno et al. 2013). Mice received intravenous injection of ^125^I‐BMIPP (5 kBq) and ^18^F‐FDG (100 kBq) via the lateral tail vein in a volume of 100 μL. ^125^I‐BMIPP was a gift from Nihon Medi‐Physics Co. Ltd. ^18^F‐FDG was obtained from batches prepared for clinical PET imaging in Gunma University. The animals were sacrificed at 2 h after injection. The isolated tissues were weighed and counted in a well‐type gamma counter (ARC‐7001, ALOKA).

### Running exercise protocol

A 2‐lane motorized rodent treadmill (MK‐680, Muromachi Kikai, Tokyo, Japan) was used to determine the endurance capacity for running (Haramizu et al. 2009; McFarlan et al. 2012). Mice were accustomed to the treadmill running according to the following 2‐day training program: day 1: 10 m/min for 15 min and 15 m/min for 15 min; day 2: 10 m/min for 10 min, 15 m/min for 10 min and 20 m/min for 10 min. On day 3, mice were subjected to a single bout of running according to the following program for measurement of endurance: 10 m/min for 5 min, 15 m/min for 5 min, 20 m/min for 5 min, 25 m/min for 10 min and 30 m/min for 30 min (Fig. 2B). Exhaustion was defined as the point at which mice spent more than 3 s on the electric shocker without attempting to resume running. Total running distance was calculated for each mouse. There was a 2‐day interval at least between each exercise.

### Measurement of blood metabolites (Syamsunarno et al. 2014; Putri et al. 2015)

Blood glucose was measured by glutest sensor (Sanwa Kagaku, Aichi, Japan). Serum levels of triglyceride (Triglyceride E‐test, Wako Chemical, Osaka), NEFA (NEFA C‐test Wako Chemical), glycerol (Free Glycerol colorimetric/Fluorometric assay kit, Biovision, USA), *β*‐hydroxybutyrate (EnzyChrom Ketone Body Assay Kit, BioAssay Systems, CA), and lactate (Lactate colorimetric Assay Kit II, Biovision) were measured according to the manufacturer's protocols.

### Glycogen and triacylglycerol measurements in liver and skeletal muscle (Syamsunarno et al. 2014; Putri et al. 2015)

Tissues were powder‐pulverized in liquid nitrogen, and a 10 mg sample was homogenized in distilled water and boiled at 99°C. After centrifugation, the supernatants were collected to measure the glycogen concentration (BioVision). For TG measurement, tissues were homogenized in RIPA buffer (50 mmol/L Tris‐HCl, pH 7.4, 1% NP40, 0.25% Na‐deoxycholate, 150 mmol/L NaCl and 1 mmol/L EDTA) and centrifuged at 18,000*g* for 10 min at 4°C. Lipids in the supernatant were extracted with methanol/chloroform (1:2), evaporated with N_2_ and dissolved in isopropanol. TG content was measured by the Triglyceride E‐test Wako (Wako Chemical) and normalized by protein concentration.

### Pyruvate challenge test (Syamsunarno et al. 2013)

After a 24‐h fast, pyruvate (2 g/kg body weight, Sigma) was injected intraperitoneally. Blood was taken from the tail vein to measure blood glucose levels using the glutest sensor (Sanwa Kagaku) at the indicated time points.

### RNA isolation and quantitative real‐time polymerase chain reaction analysis

Total RNA isolation and quantitative real time‐PCR were performed as described previously (Syamsunarno et al. 2013). The expression levels of the target genes were normalized to the GADPH (glyceraldehyde 3‐phosphate dehydrogenase) mRNA level. The ready‐to‐use gene‐specific primers for cDNA (perfect real time primer) were purchased from Takara Bio Inc. (Shiga, Japan).

### Immunofluorescence and histochemical analysis (Bloemberg and Quadrilatero 2012)

Whole soleus was removed and a portion of the entire circumference around the mid‐belly was used. Muscles were embedded in O.C.T. compound (Tissue‐Tek), frozen in liquid nitrogen‐cooled hexane, stored at −80°C, and cut into 10 *μ*m thick cryosections with a cryostat (Leica CM3050S, Germany) maintained at −20°C. Immunofluorescence analysis of myosin heavy chain (MHC) isoform expression was performed with primary antibodies against MHCI (BA‐F8) and MHCIIa (SC‐71), both of which were purchased from the Developmental Studies Hybridoma Bank (University of Iowa). Secondary antibodies (Alexa Fluor 350 IgG2b for MCH1 and Alexa Fluor 555 IgG1 for MHCIIa) and Alexa Fluor 488‐conjugated wheat germ agglutinin were purchased from Invitrogen. Muscle fibers negative for MHCI and MHCIIa were recognized as combination of type IIb and IIx fibers. Percentages of fiber types were calculated by counting muscle fibers positive or negative for each MHC isoforms.

### Statistical analysis

Statistical analysis of the data was performed with IBM SPSS (version 24 for Windows, IBM, NY). The data are presented as mean ± standard deviation. Student's *t*‐test was performed for 2 groups’ comparison. Two‐way analysis of variance (ANOVA) was used to analyze effects of feeding status (fed or fasted) on tracer uptake and running distance between WT and CD36^−/−^ (CD36 genotype). Three‐way ANOVA was used to determine whether there were any interaction effects on levels of metabolites and gene expression among CD36 genotype, the feeding status and exercise. A *P*‐value < 0.05 was considered to be statistically significant.

## Results

### Effects of fasting on FA and glucose uptake in the heart and skeletal muscles

**Figure 1:**
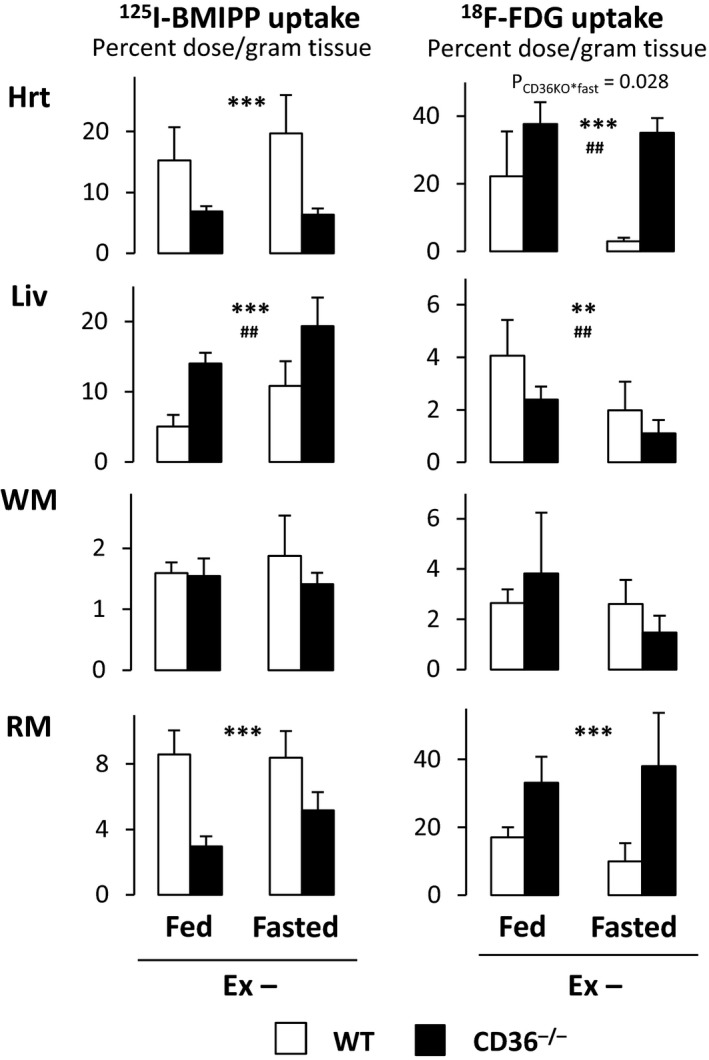
Uptake of NEFA tracer and glucose tracer by the heart, liver, and skeletal muscle in the fed or fasted state. Two hours after injection of ^125^I‐BMIPP and ^18^F‐FDG with or without a 24‐h fast, mice were sacrificed to isolate tissues and determine uptake of ^125^I‐BMIPP and ^18^F‐FDG by the heart (Hrt), liver (Liv), white/glycolytic skeletal muscle (WM, quadriceps femoris) and red/oxidative skeletal muscle (RM, soleus). *n* = 6. Ex, exercise. **P < 0.01, ***P < 0.001, main effect for genotype. ^##^ *P*<0.01, main effect for fasting. Interaction is indicated with *P*‐value in the figure if it is statistically significant.

To compare difference of FA and glucose uptake between WT and CD36^−/−^ mice in the fed and fasted states, we examined the biodistribution of the slowly oxidized FA analogue ^125^I‐BMIPP and the metabolically trapped glucose analogue ^18^F‐FDG. A significant reduction in ^125^I‐BMIPP uptake was observed in the heart and red/oxidative skeletal muscle (soleus) in CD36^−/−^ mice irrespective of feeding conditions (Fig. 1). ^125^I‐BMIPP uptake was not impaired in white/glycolytic skeletal muscle (quadriceps femoris), which mainly consumes glucose. ^18^F‐FDG uptake increased dramatically in the heart and red/oxidative skeletal muscle in CD36^−/−^ mice. Significant interaction between CD36 deficiency and fasting was observed on ^18^F‐FDG uptake of the heart (*P* = 0.028). Prolonged fasting resulted in a marked reduction in ^18^F‐FDG uptake in the heart in WT mice only whereas no change occurred in CD36^−/−^ mice, suggesting insulin‐independent glucose uptake in CD36^−/−^ mice (Iso et al. 2013). In contrast to the heart and red muscle, there was no significant difference in ^18^F‐FDG uptake in white/glycolytic skeletal muscle, strongly suggesting that an augmentation of glucose uptake occurs to compensate for a reduction in FA uptake in FA‐consuming tissues (Fig. 1). Our data support the idea that compensatory glucose use is not affected by fasting in CD36^−/−^ mice, which is in sharp contrast to WT mice. Thus, it is likely that glucose is a predominant energy substrate in CD36^−/−^ mice even during fasting.

### A significant reduction in treadmill exercise endurance in CD36^−/−^ mice in the fasted state

**Figure 2:**
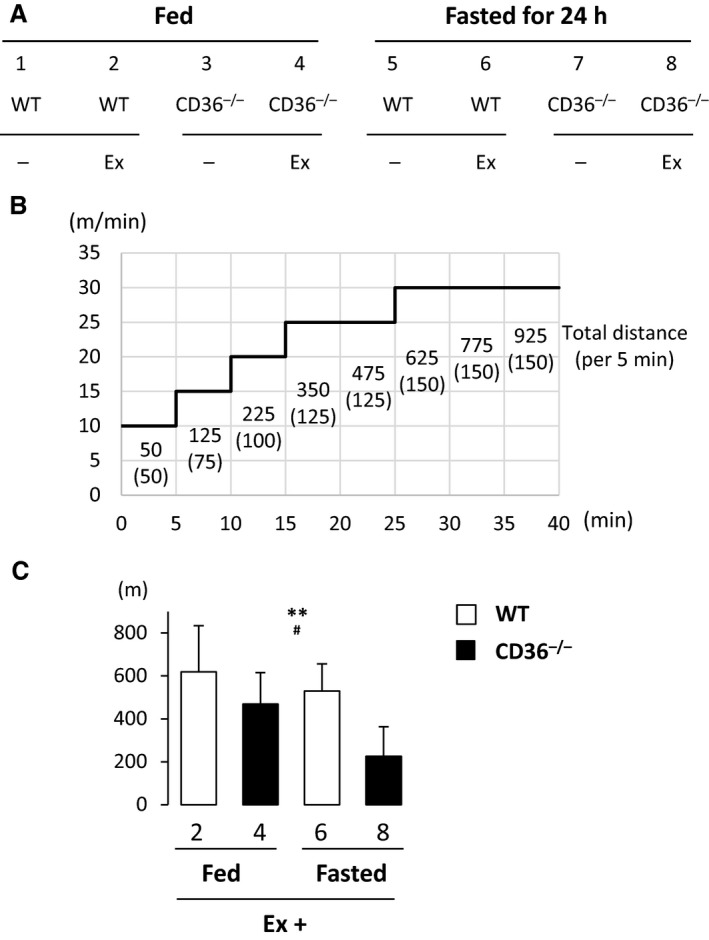
Running endurance capacity in CD36^−/−^ mice is markedly reduced after a 24‐h fast. (A) Eight groups prepared for this study. Ex, exercise. (B) The exercise protocol used in this study. See the method in detail. (C) Running distance for each group. *n* = 6. **P < 0.01, main effect for genotype. ^#^ *P* < 0.05, main effect for fasting.

We next tested our initial hypothesis that running endurance is reduced in CD36^−/−^ mice in the fasted state. Running exercise was started at the speed of 10 meter/min for 5 min, and the speed was progressively increased until mice were exhausted (Fig. 2A and B). As shown in Figure 2C, running distance was significantly decreased by CD36 deficiency and fasting, resulting in the shortest distance in CD36^−/−^ mice in the fasted state.

### Effects of exercise on blood glucose, lipids, ketone body, and lactate in fed and fasted mice

**Figure 3:**
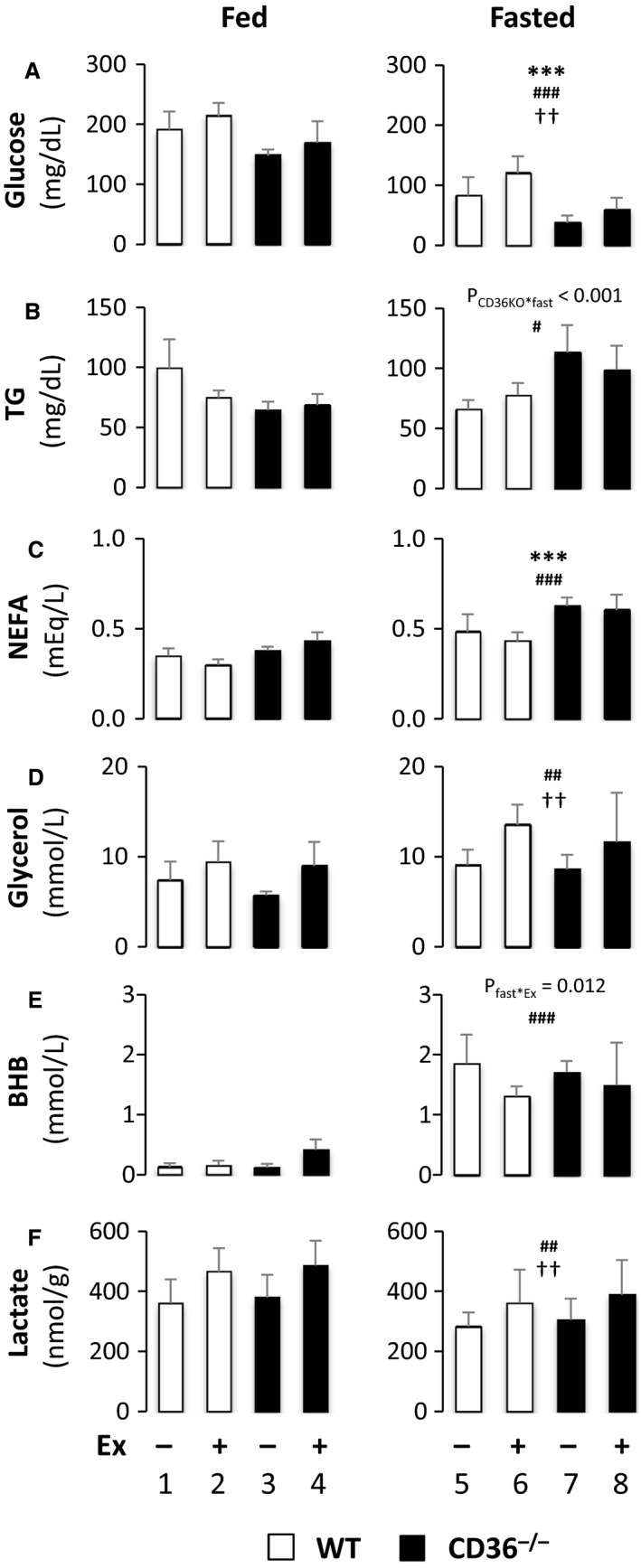
Serum levels of metabolites. Blood was collected with or without treadmill exercise to measure serum levels of glucose (A), TG (B), NEFA (C), glycerol (D), β‐hydroxybutyrate (BHB) (E), and lactate (F) in the fed or fasted state. TG, triacylglycerol; NEFA, non‐esterified fatty acid; BHB, *β*‐hydroxy butyrate. *n* = 6. ***P < 0.001, main effect for genotype. ^#^ *P* < 0.05, ^##^ *P* < 0.01, ^###^ *P* < 0.001, main effect for fasting. ^††^ *P* < 0.01, main effect for exercise. Interaction is indicated with *P*‐value in the figure if it is statistically significant.

We sought for the reasons of reduced endurance in fasted CD36^−/−^ mice. We first examined biochemical parameters with and without treadmill exercise in the fed and fasted states. A reduction in serum glucose levels by fasting was enhanced in CD36^−/−^ mice (Fig. 3A). An increase in serum levels of NEFAs by fasting was elevated in CD36^−/−^ mice (Fig. 3C), which was consistent with reduced FA uptake and oxidation by peripheral tissues in CD36^−/−^ mice. Glycerol levels were comparably elevated by fasting and exercise in WT and CD36^−/−^ mice (Fig. 3D), which suggests that lipolysis was similarly induced in both mice. Serum levels of ketone body (β‐hydroxybutyrate, BHB) were similarly elevated by fasting in WT and CD36^−/−^ mice (Fig. 3E). There was significant interaction between fasting and exercise (*P*_fast*Ex_ = 0.012), suggesting that ketone body utilization exceeded ketogenesis during exercise in the fasted state. Serum levels of lactate were similarly induced by exercise (Fig. 3F), suggesting that anaerobic glycolysis occurred comparably. Given that glucose seems to be crucial energy substrate for CD36^−/−^ mice even during fasting (Fig. 1), our data suggest that accelerated hypoglycemia due to CD36 deficiency and fasting (Fig. 3A) could cause detrimental effects on exercise endurance of skeletal muscle in fasted CD36^−/−^ mice.

### Effects of exercise on energy storage in liver and skeletal muscle

**Figure 4:**
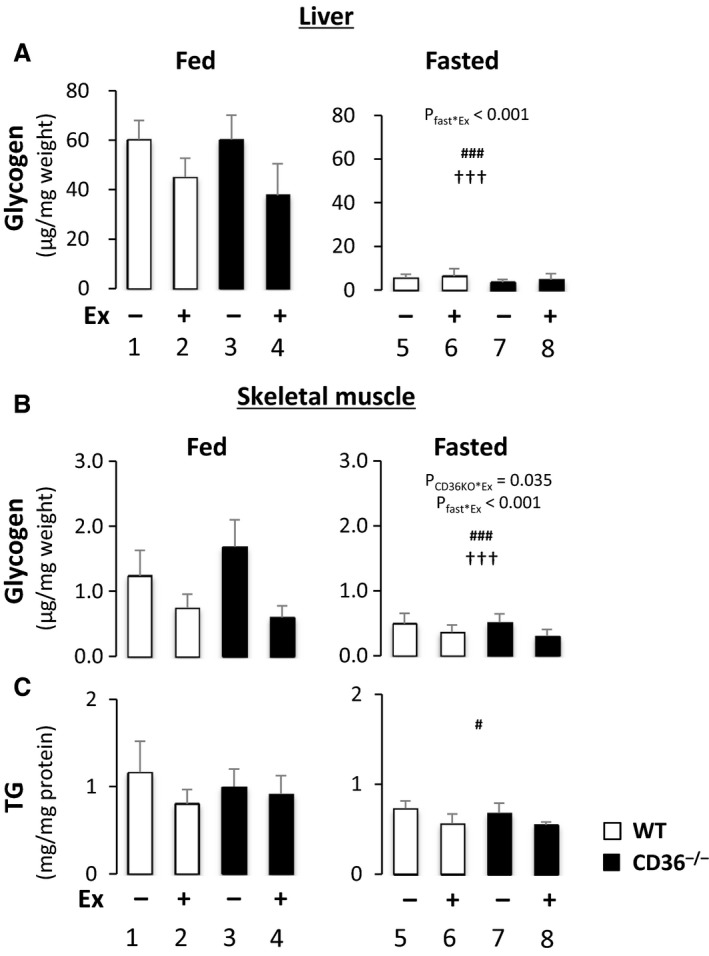
Storage of glycogen and TG in liver and skeletal muscle. Liver and quadriceps femoris muscle were isolated with or without treadmill exercise in the fed or fasted state to measure glycogen and TG content. (A) Glycogen content in liver. (B) Glycogen content in quadriceps femoris. (C) TG content in quadriceps femoris. TG content was normalized by protein concentration. *n* = 6. TG, triacylglycerol. ^#^ *P* < 0.05, ^###^ *P* < 0.001, main effect for fasting. ^†††^ *P* < 0.001, main effect for exercise. Interaction is indicated with *P*‐value in the figure if it is statistically significant.

We next examined glycogen and TG storage in liver and skeletal muscle. Glycogen levels in liver and skeletal muscle were significantly reduced by fasting, leading to the reductions of their baseline levels, which resulted in blunted responses to exercise (Fig. 4A and B). The reduction in glycogen by exercise in liver and skeletal muscle was blunted in the fasted state in both WT and CD36^−/−^ mice (Fig. 4A, *P*_fast*Ex_ < 0.001 and B, *P*_fast*Ex_ < 0.001), suggesting near depletion of glycogen after a 24‐h fast prior to exercise. TG content in skeletal muscle was reduced by fasting, but not by exercise (Fig. 4C). These findings suggest that glycogen storage in liver and skeletal muscle is important energy substrate for an acute bout of exercise, but it becomes unavailable after prolonged fasting prior to exercise.

### Gluconeogenesis and expression of the related genes in liver

**Figure 5:**
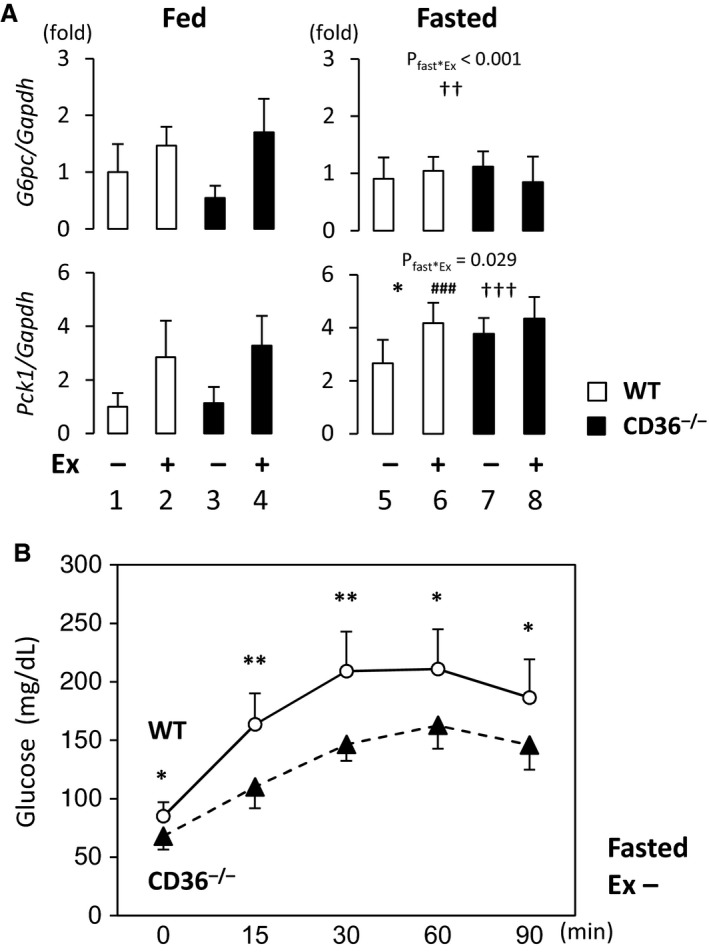
Relative supply of glucose via gluconeogenesis was reduced after a 24‐h fast in CD36^−/−^ mice. (A) Liver was isolated with or without treadmill exercise in the fed or fasted state. The total RNA was extracted for quantitative real‐time PCR. *G6pc*, glucose‐6‐phosphate catalytic subunit; *Pck1*, phosphoenolpyruvate carboxykinase 1; *Gapdh*, glyceraldehyde 3‐phosphate dehydrogenase. *n* = 6. *P < 0.05, main effect for genotype. ^###^ *P* < 0.001, main effect for fasting. ^††^ *P* < 0.01, ^†††^ *P* < 0.001, main effect for exercise. Interaction is indicated with *P*‐value in the figure if it is statistically significant. (B) Elevation of serum glucose levels via gluconeogenesis by the pyruvate challenge test. After a 24‐h fast, pyruvate (2 g/kg) was intraperitoneally injected. Blood was taken from the tail vein to measure blood glucose levels at the indicated time points. *n* = 6. *P < 0.05; **P < 0.01.

The expression of key enzymes for gluconeogenesis, glucose‐6‐phosphate catalytic subunit (*G6pc*) and phosphoenolpyruvate carboxykinase (*Pck1*), was significantly induced by exercise (Fig. 5A). The expression of *Pck1* was also induced by fasting (Fig. 5A). An increase in expression levels of *G6pc* and *Pck1* by exercise was blunted in the fasted state (Fig. 5A, *G6pc*,* P*_fast*Ex_ < 0.001; *Pck1*,* P*_fast*Ex_ = 0.029). To examine the extent of gluconeogenesis after a 24‐h fast, a pyruvate challenge test was performed to measure the blood glucose in response to intra‐peritoneal administration of pyruvate (a major gluconeogenic substrate). As shown in Figure 5B, CD36^−/−^ mice were compromised in the pyruvate challenge irrespective of preserved expression of gluconeogenesis‐related genes during fasting. A reduction in blood glucose after the pyruvate challenge may be due to accelerated glucose consumption in the heart and skeletal muscle at least in part. These findings suggest that reduced glucose supply relative to its demand also contribute to severe hypoglycemia in fasted CD36^−/−^ mice.

### Expression of genes associated with muscle energy metabolism in skeletal muscle

**Figure 6:**
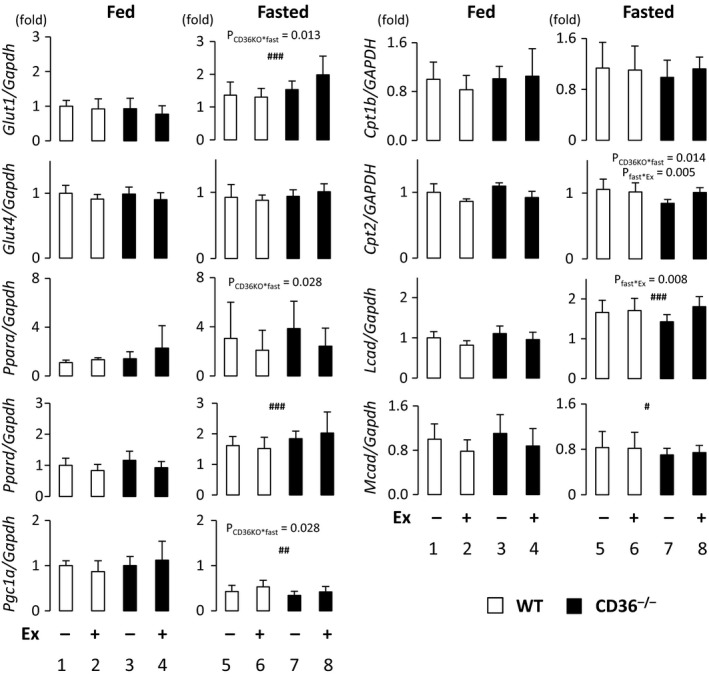
Expression of genes associated with glucose and fatty acid metabolism in skeletal muscle. Quadriceps femoris muscle was isolated with or without treadmill exercise in the fed or fasted state. The total RNA was extracted for quantitative real‐time PCR. *Ppara*, peroxisome proliferator activated receptor *α*;* Ppard*, Ppar *δ*,* gc1a*, PPAR *γ* coactivator 1 *α*;* Cpt1b/2*, carnitine palmitoyltransferase 1b/2; *Mcad*, medium‐chain acyl‐CoA dehydrogenase; *Lcad*, long‐chain acyl‐CoA dehydrogenase. *n* = 6. ^#^ *P* < 0.05, ^##^ *P* < 0.01, ^###^ *P* < 0.001, main effect for fasting. Interaction is indicated with *P*‐value in figure if it is statistically significant.

We next examined levels of mRNAs for a variety of genes relevant to glucose and FA metabolisms in skeletal muscle. Expression of glucose transporters, *Glut1* and *Glut4*, was not significantly different between WT and CD36^−/−^ mice (Fig. 6). Expression of mRNA for peroxisome proliferator‐activated receptor *α* (*Ppara)*,* Ppard* and their co‐activator, PPAR*γ*‐coactivator‐1 alpha (*Pgc1a*), which are central regulators involved in FA uptake and oxidation, was not significantly changed between WT and CD36^−/−^ mice (Fig. 6). Consistently, the expression of the battery of genes regulated by PPAR/PGC‐1 complex was not changed, either. Those include the genes for FA oxidation‐related mitochondrial enzymes, *Cpt1b*,* Cpt2*,* Mcad*, and *Lcad*. Thus, there seems to be no significant difference regarding mRNA expression of genes relevant to glucose and FA metabolism between WT and CD36^−/−^ mice irrespective of fasting and exercise.

### No change in isoforms of MHC in oxidative skeletal muscle fiber

**Figure 7:**
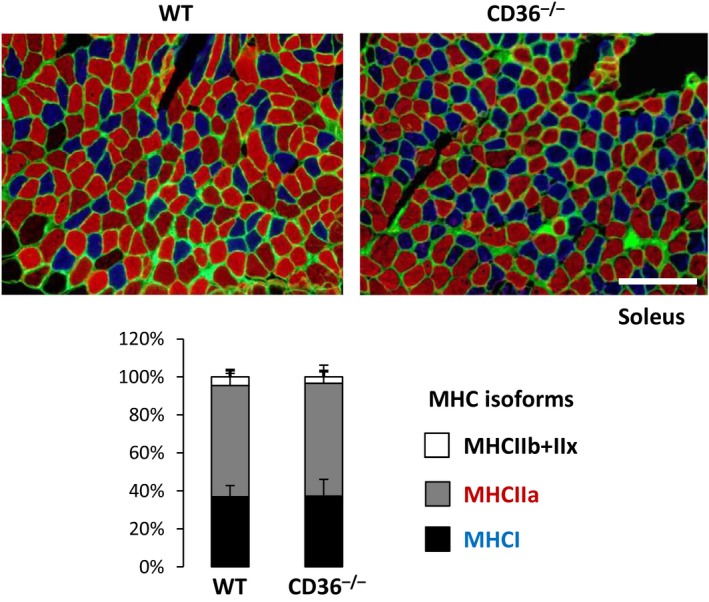
Composition of fiber types in oxidative skeletal muscle was comparable between WT and CD36^−/−^ mice. Soleus muscle was isolated from WT and CD36^−/−^ mice to estimate expression of MHC isoforms by immunofluorescense. Muscle fibers negative for MHCI and MHCIIa were represented as combination of type IIb and IIx fibers. Bar graph shows percentage of each MHC isoforms. MHC, myosin heavy chain. *n* = 6. Scale bar, 100 *μ*m.

Skeletal muscle fibers are classified into type I (oxidative/slow‐twitch) or type II (glycolytic/fast‐twitch) fibers (Egan and Zierath 2013). They display marked differences regarding contraction, metabolism, and susceptibility to fatigue. Type I fibers, which mainly use oxidative metabolism for energy production, are mitochondria‐rich and fatigue‐resistant. Type II fibers comprise three subtypes, IIa, IIb, and IIx. Type IIb fibers, which mainly rely on glycolytic metabolism, have the lowest levels of mitochondrial content and are susceptible to fatigue. The oxidative and contraction functions of type IIa lie between type I and IIb. The function of type IIx is similar to type IIb. It is known that adult skeletal muscle shows plasticity and can undergo conversion between different fiber types in response to exercise training, modulation of motor neuron activity and metabolic overload such as obesity (Wang et al. 2004). Therefore, we questioned whether a substrate shift from FA to glucose observed in oxidative skeletal muscle (i.e., soleus) induces phenotypic changes of muscle fibers from type I to type II types, which might cause reduced endurance capacity for treadmill exercise. Muscle fiber types were determined by expression of MHC isoforms by immunofluorescence as described previously (Bloemberg and Quadrilatero 2012). We found that composition of MHC isoforms in soleus muscle was comparable between WT and CD36^−/−^ mice (Fig. 7), suggesting that substrate shift from FA to glucose in oxidative skeletal muscle does not induce phenotypic conversion of muscle fiber types.

## Discussion

**Figure 8:**
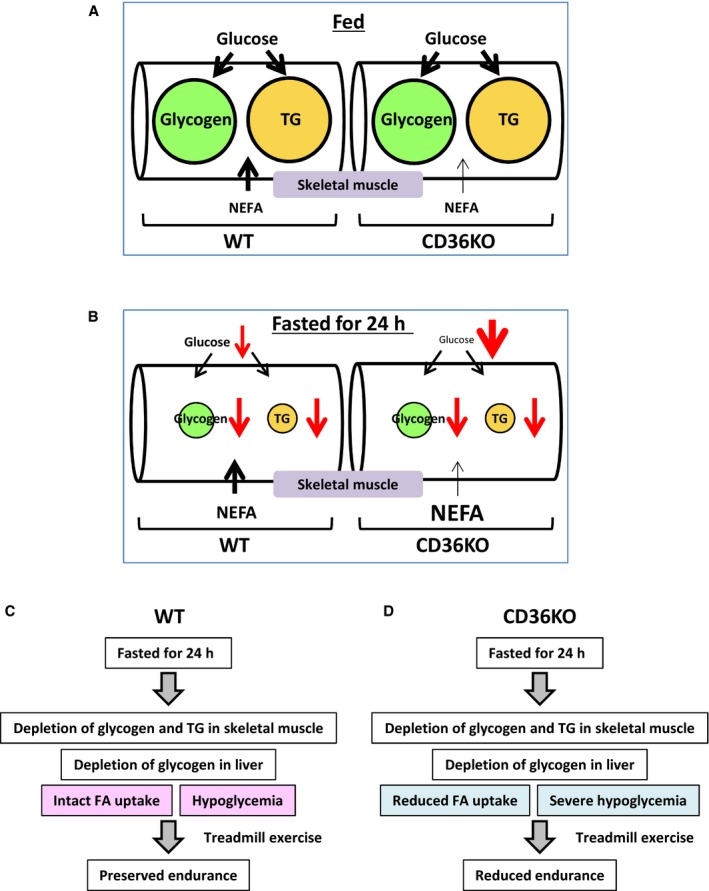
Reduced FA uptake and severe hypoglycemia cause reduced exercise performance in CD36^−/−^ mice during prolonged fasting. (A) In the fed state, intramuscular energy storage are sufficient for an acute bout of exercise in both WT and CD36^−/−^ mice. Although FA uptake is impaired in red skeletal muscle in CD36^−/−^ mice, it does not affect exercise endurance when exercise intensity is high and exercise duration is short. (B) In the fasted state, intramuscular glycogen and TG are nearly depleted in both WT and CD36^−/−^ mice. FA uptake is continuously impaired in red skeletal muscle in CD36^−/−^ mice even during fasting. Hypoglycemia is accelerated in CD36^−/−^ mice because of compensatory glucose use in the heart and red skeletal muscle. (C and D) After prolonged fasting, major energy substrates for exercise are circulating NEFA and glucose via liberation from TG of adipose tissue and hepatic gluconeogenesis, respectively. (C) In fasted WT mice, circulating NEFA and glucose are constantly suppled to skeletal muscle, which results in preserved endurance in WT mice. (E) In fasted CD36^−/−^ mice, FA uptake is reduced in red skeletal muscle although serum NEFA levels are elevated. Glucose supply to both white and red muscle is reduced due to severe hypoglycemia. Because compensatory uptake of glucose does not occur in white muscle, energy insufficiency is more enhanced in white muscle. Thus, reduced FA uptake and severe hypoglycemia could cause reduced endurance. TG, triacylglycerol; NEFA, non‐esterified fatty acid. Size of characters and arrows indicates relative amount of energy substrates.

In this study, we demonstrated an indispensable role of CD36 in exercise performance during prolonged fasting. After a 24‐h fast, glycogen in liver and skeletal muscle was depleted both in WT and CD36^−/−^ mice. In CD36^−/−^ mice, glucose utilization continued to be enhanced in the heart and red/oxidative skeletal muscle and glucose supply relative to its demand was likely to be diminished, which led to accelerated hypoglycemia. Furthermore, FA uptake was reduced in red/oxidative skeletal muscle due to CD36 deficiency. Consequently, available energy substrates in serum and skeletal muscle for exercise performance were very limited in CD36^−/−^ skeletal muscle during prolonged fasting (Fig. 8B and D), which could cause enhanced reduction in endurance capacity. Thus, abnormal uptake and distribution of energy substrates cause impaired nutrient homeostasis in the fasted state, resulting in early exhaustion upon strenuous exercise.

It has been thought that liver and muscle glycogen is a key energy substrate for endurance capacity. In this study, we propose that energy substrates supplied from circulation can be major contributing factors for endurance capacity when intramuscular energy storage is depleted (Fig. 8B, C and D). In fasted WT and CD36^−/−^ mice, muscle glycogen and TG were nearly depleted prior to exercise (Figs. 4B, C and 8C, D). Major difference of energy sources for exercise between them were two factors, FA uptake by oxidative muscle and blood glucose levels (Fig. 8C and D). Thus, our data suggest that the difference of energy supply from circulation can also be factors to determine endurance capacity. It remains unsolved, however, which substrate is more influential for exercise endurance in this study. In humans, it is reported that whole muscle consists of 54% of type I, 32% of type IIa and 13% of type IIx fibers (Egan and Zierath 2013). Bloemberg and Quadrilatero (2012) reported that typical red/oxidative muscle (soleus) is comprised of 35% of type I, 60% of type IIa and ~5% of type IIb and IIx fibers in mice. These findings suggest that type I fiber is the most predominant component in human skeletal muscle, but not in mice, which raise a next assumption that whole muscle in mice may prefer glucose to FA compared to humans. Therefore, accelerated hypoglycemia during fasting may influence endurance capacity more severely than reduced FA uptake by oxidative muscle in fasted CD36^−/−^ mice. It is warranted to address which factor, FA or glucose supply from circulation, contributes more to endurance capacity during fasting in the future.

McFarlan et al. (2012) demonstrated that exercise performance was reduced by 44% in CD36^−/−^ mice compared to WT (180 min vs. 100 min) when running at 17 m/min (78% VO_2_max, aerobic exercise). Consistent with it, we also showed reduced endurance in CD36^−/−^ mice after a 24‐h fast (450 m vs. 200 m). Three out of six fasted CD36^−/−^ mice showed exhaustion when running at 15 m/min (submaximal aerobic level). A common finding in these experiments above is that glycogen storage in liver and skeletal muscle was depleted when they stopped running, supporting the notion that liver and skeletal muscle glycogen is a key nutrient for endurance capacity. In contrast to reduced endurance in the fasted state, however, there was no significant difference when running at final speed of 30 m/min in the fed state. Comparable elevation of lactate after exercise suggested that anaerobic glycolysis similarly occurred in both mice. Glycogen levels in liver and skeletal muscle were comparable and above empty levels when compared to those after a 24‐h fast, suggesting that a reason for exhaustion at high‐intensity exercise was probably due to muscle fatigue, not energy depletion. These findings raise an interesting possibility that exercise performance is not impaired in CD36^−/−^ mice when exercising at anaerobic levels in the fed state, when glycogen storage is sufficient.

CD36 deficiency has been also reported in humans (Fukuchi et al. 1999; Tanaka et al. 2001). In patients with type I CD36 deficiency (homozygote or compound heterozygote of loss‐of‐function mutation), FA uptake is markedly reduced with a robust increase in glucose uptake in the heart‐like CD36^−/−^ mice. Although several reports documented the association of CD36 deficiency to metabolic diseases in humans (Miyaoka et al. 2001; Kuwasako et al. 2003), there is no report showing that CD36 deficiency impairs endurance exercise capacity. Further work should be warranted to prove the role of CD36 in exercise capacity of skeletal muscle in humans.

## Conclusion

Our data provide the experimental evidence to demonstrate that FA uptake through CD36 is crucial for exercise endurance during fasting, and imply that CD36 is indispensable for nutrient homeostasis when FA demand is increased and nutrient availability is limited.

## Conflict of Interest

None declared.
